# Case Report: p40*^phox^* deficiency underlying pediatric-onset systemic lupus erythematosus

**DOI:** 10.3389/fped.2024.1425874

**Published:** 2024-08-20

**Authors:** Alejandro Nieto-Patlán, Natalia S. Fernández Dávila, Yuqing Wang, Michelle Zelnick, Eyal Muscal, Martha Curry, James R. Lupski, Steven M. Holland, Bo Yuan, Douglas B. Kuhns, Tiphanie P. Vogel, Ivan K. Chinn

**Affiliations:** ^1^ Division of Immunology, Allergy, and Retrovirology, Department of Pediatrics, Baylor College of Medicine and Texas Children’s Hospital, Houston, TX, United States; ^2^ Department of Integrative Physiology, Baylor College of Medicine, Houston, TX, United States; ^3^ Division of Rheumatology, Department of Pediatrics, Baylor College of Medicine and Texas Children’s Hospital, Houston, TX, United States; ^4^ Department of Molecular and Human Genetics, Baylor College of Medicine, Houston, TX, United States; ^5^ Human Genome Sequencing Center, Baylor College of Medicine, Houston, TX, United States; ^6^ Texas Children's Hospital, Houston, TX, United States; ^7^ Laboratory of Clinical Immunology and Microbiology, National Institute of Allergy and Infectious Diseases, National Institutes of Health, Frederick, MD, United States; ^8^ Neutrophil Monitoring Laboratory, Clinical Services Program, Leidos Biomedical Research Inc., Frederick National Laboratory for Cancer Research, Frederick, MD, United States

**Keywords:** p40*^phox^* deficiency, NADPH oxidase complex, *NCF4*, reactive oxygen species, systemic lupus erythematosus, inborn error of immunity, pediatric SLE

## Abstract

**Introduction:**

Systemic lupus erythematosus is a multi-faceted autoimmune disorder of complex etiology. Pre-pubertal onset of pediatric systemic lupus erythematosus (pSLE) is uncommon and should raise suspicion for a genetic driver of disease. Autosomal recessive p40*^phox^* deficiency is a rare immunologic disorder characterized by defective but not abolished NADPH oxidase activity with residual production of reactive oxygen species (ROS) by phagocytic cells.

**Case presentation:**

We report the case of a now 18-year-old female with pSLE onset at 7 years of age. She presented with recurrent fever and malar rash. Aspects of her immune dysregulation over time have included typical pSLE features including production of autoantibodies, hematologic manifestations, and hypocomplementemia, as well as chronic suppurative skin lesions and recurrent infections. Genetic analysis revealed biallelic pathogenic variants in *NCF4* resulting in p40*^phox^* deficiency. Comprehensive NADPH oxidase activity studies confirmed significantly decreased production of reactive oxygen species, confirming the cellular phenotype seen in p40*^phox^* deficient patients.

**Conclusions:**

Here, we present a patient with pSLE harboring biallelic variants in *NCF4*. Our patient represents a unique clinical presentation of severe onset autoimmunity in the setting of a rare inborn error of immunity affecting NADPH oxidase activity. This case underscores the need to consider genetic causes of pSLE in cases of pre-pubertal onset and atypical disease.

## Introduction

Phagocyte oxidase subunit p40 (p40*^phox^*) deficiency is an inborn error of immunity characterized by impaired production of reactive oxygen species (ROS) by phagocytic cells. Unlike classic chronic granulomatous disease (CGD), individuals with p40*^phox^* deficiency do not suffer from invasive bacterial and fungal infections, and their phagocytic cells exhibit residual ROS production (1). However, despite the residual ROS production, p40*^phox^* deficient patients can develop immune dysregulation, leading to various clinical manifestations, including superficial infections and inflammatory phenotypes, such as inflammatory bowel (IBD) disease and cutaneous lupus (1), and immune-mediated thrombocytopenia (ITP) (2). In adults, individuals with hypomorphic mutations in *NCF2* (p67*^phox^*) and female carriers of *CYBB* (gp91*^phox^*) mutations can develop multi-organ autoimmunity such as systemic lupus erythematosus (SLE) (3, 4).

SLE is a multisystem autoimmune disease with heterogeneous presentations including systemic and cutaneous inflammation, various organ system involvement such as arthritis, nephritis, and immune-mediated cytopenias, and elevated circulating autoantibodies. Pediatric SLE (pSLE) refers to cases wherein the onset of disease occurs under the age of 18 years, and such patients are at increased risk of disease-related organ damage and treatment-related morbidity over time (5, 6). While the underlying pathophysiology of SLE is complex, a small percentage of pSLE cases are the result of inborn errors of immunity associated with monogenic defects. Several genes, including ones resulting in complement deficiencies and interferonopathies, have been implicated in lupus predisposition (7, 8).

Here we report a patient with pSLE and autosomal recessive p40*^phox^* deficiency, identified through exome sequencing (ES). This patient presented originally at 7 years old with malar rash and developed severe clinical manifestations, including refractory ITP and recurrent infections. Functional evaluation of NADPH oxidase activity through the dihydrorhodamine 1,2,3 (DHR) assay confirmed the cellular phenotype seen in p40*^phox^* deficient patients with defective, but not abolished, ROS production. Our study expands the clinical spectrum of p40*^phox^* deficiency and highlights the importance of considering this genetic disorder in the differential diagnosis of unresolved pSLE cases.

## Case description

**Figure 1:**
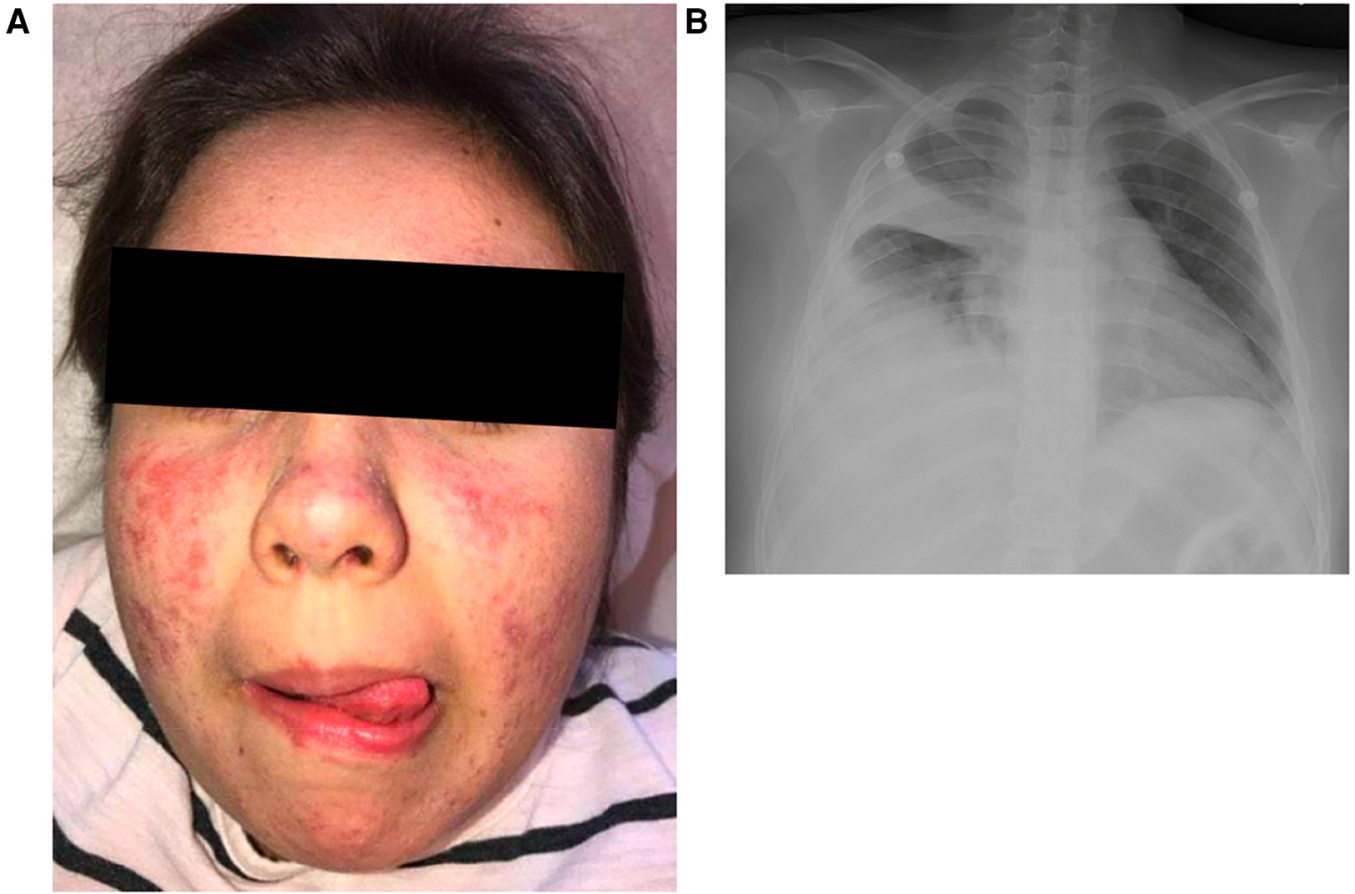
Clinical findings. **(A)** Photosensitive malar rash sparing the nasolabial folds. **(B)** Chest imaging showing right middle and lower lobe consolidation and parapneumonic effusion and right upper lobe atelectasis at the time of presentation with acute hypoxemic respiratory failure requiring management with bilevel positive airway pressure.

A now 18-year-old female was diagnosed with pSLE at the age of 7 years. Her initial presentation included sterile fevers, malar rash (Figure 1A), the presence of antinuclear antibodies (ANA), autoimmune hemolytic anemia, hypocomplementemia, and anti-phospholipid antibodies (lupus anticoagulant)—meeting classification criteria for SLE according to both the European League Against Rheumatism/American College of Rheumatology (EULAR/ACR) and the Systemic Lupus International Collaborating Clinics (SLICC) criteria (9, 10). She received high-dose intravenous methylprednisolone and was treated with hydroxychloroquine and mycophenolate mofetil.

Following her initial pSLE diagnosis, additional autoantibodies developed, including anti-Smith, and she developed recurrent and refractory ITP. B-cell phenotyping revealed normal proportions of naïve and memory B cells with no increased CD21^low^CD38^low^ B cells. In the setting of positive autoantibodies, her ITP was managed with prednisone, azathioprine, and rituximab. Her refractory ITP has required serial courses of rituximab over time for control. She has had no evidence of nephritis.

Additional relevant medical history post-pSLE diagnosis includes multiple bacterial urinary tract infections including one associated with presumed sepsis, acyclovir-responsive blepharitis, persistent right eyelid hordeolum, facial folliculitis, a consolidative pneumonia with parapneumonic effusion requiring drainage (Figure 1B), and fungal urinary tract infections secondary to *Candida* spp. Given her recurrent infections out of proportion to a typical pSLE course, additional immunologic studies were performed while she was already receiving immune suppression. She was found to have mildly elevated immunoglobulin levels and T cell and NK cell lymphopenia. At age 15 she developed skin abscesses on her neck, axillae, and chest that were successfully treated with oral antibiotics. She has never developed signs or symptoms suggestive of IBD.

## Diagnostics

**Figure 2:**
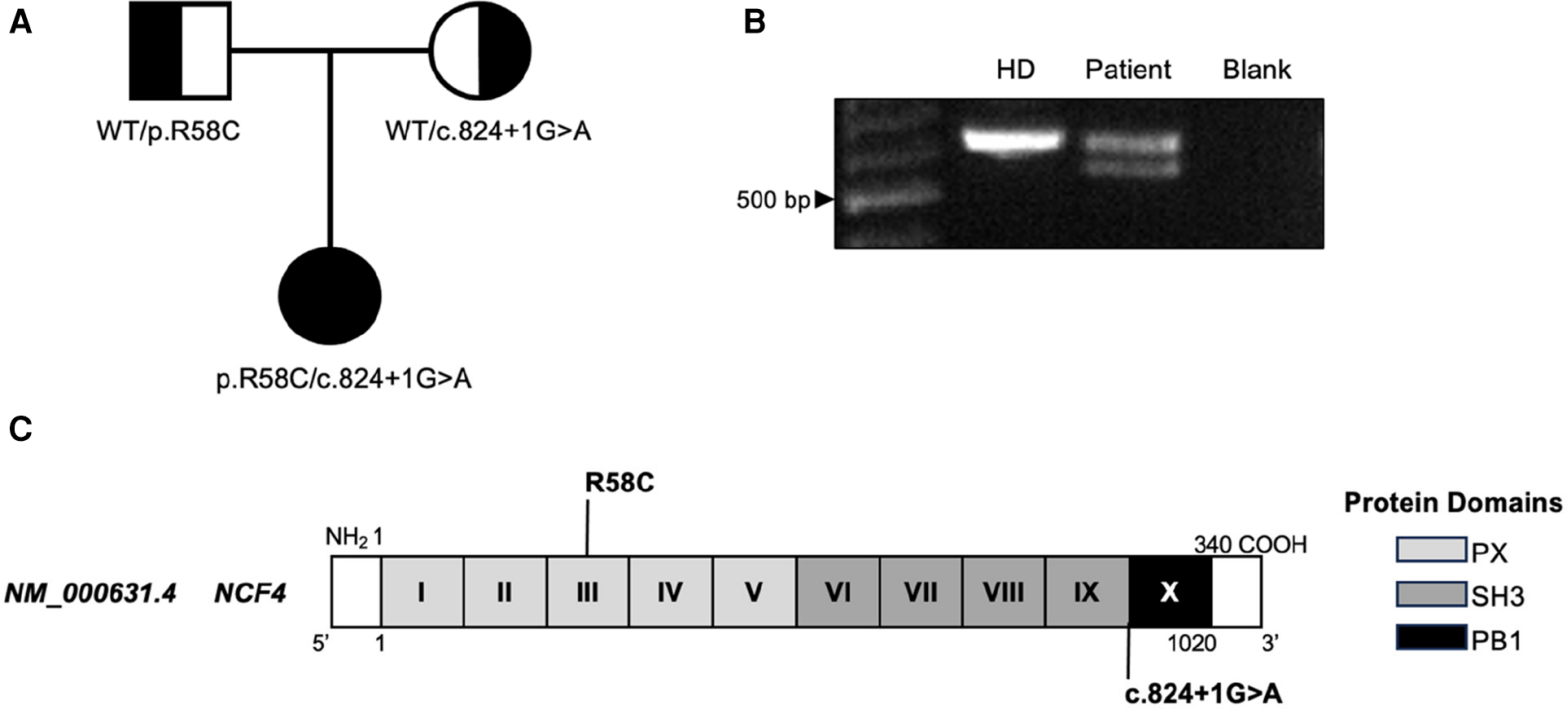
NCF4. **(A)** Familial segregation of *NCF4* variants. **(B)** RT-PCR of *NCF4* mRNA flanking exon 9 in the patient and a control. **(C)** Gene structure of *NCF4* and protein domains of p40*^phox^* with patients’ variants indicated. bp, base pair; HD, healthy donor.

Five years after her pSLE diagnosis, she underwent research trio ES as part of an institutional genotype screening program for patients with pSLE. Variant analysis ruled out variants in known genes associated with monogenic SLE, including genes related to complement deficiencies, nucleic acid sensing, type I interferonopathies, and tolerance defects (8). However, the patient was found to have biallelic variants in *NCF4*, each inherited from a different parent (Figure 2A). The first variant identified, p.R58C, has previously been described as a pathogenic variant conferring p40*^phox^* deficiency (1). The second variant, c.824+1G>A, is novel and was predicted to cause loss of the donor splice site. Confirmation of alternative splicing was assessed by RT-PCR using mRNA from patient peripheral blood mononuclear cells. The presence of an alternate transcript with shorter length was observed (Figure 2B); Sanger sequencing of the fragment confirmed an alternate splicing event resulting in in-frame skipping of all of exon 9. Exon 9 partially encodes the SH3 domain (Figure 2C) essential for p40*^phox^* binding to other NADPH subunits, such as p67*^phox^* (11).

**Figure 3:**
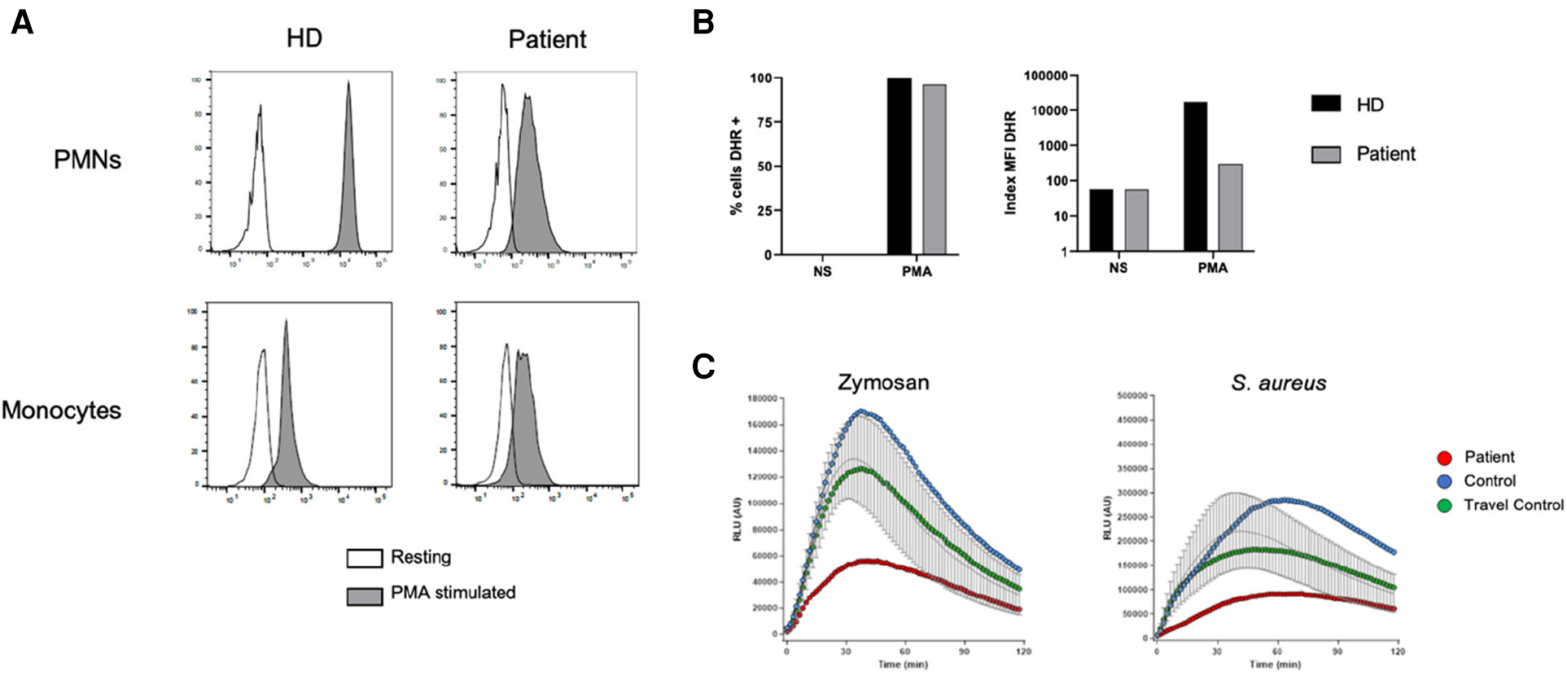
Functional studies. **(A)** DHR assay in neutrophils (top) and monocytes (bottom) upon PMA stimulation in the patient and a control. **(B)** Percentage (left) and Index MFI (right) of cells able to oxidize the DHR. **(C)** H_2_O_2_ release from patient and control neutrophils following different stimuli. DHR, dihydrorhodamine 1,2,3; HD, healthy donor; MFI, mean florescence intensity; NS, non-stimulated; PMA, phorbol 12-myristate 13-acetate; PMN, polymorphonuclear cell; RLU, relative light units.

**Figure 4:**
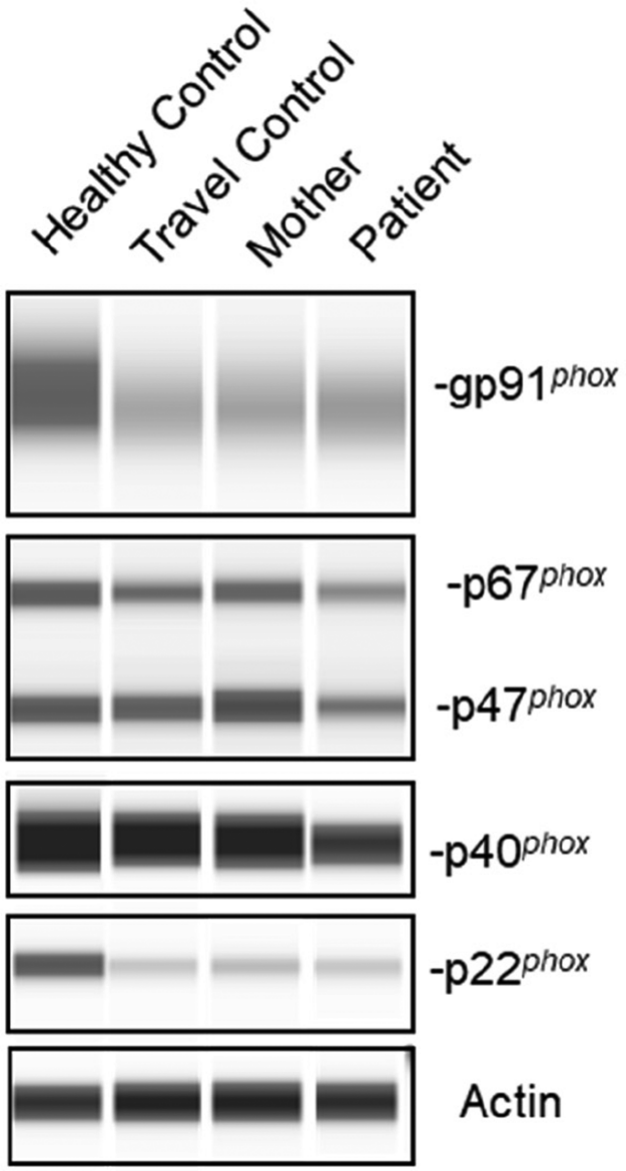
Protein expression of NADPH oxidase subunits. Protein expression of the NADPH subunits in neutrophil lysates from controls, the patient's mother (heterozygous for the *NCF4* c.824+1G>A variant) and the patient. Actin protein was used as loading control.

To confirm p40*^phox^* deficiency, the patient underwent comprehensive NAPDH oxidase studies. The DHR oxidation assay showed defective oxidation upon PMA stimulation in patient neutrophils, although all neutrophils and monocytes were capable of oxidizing DHR (Figure 3A). However, patient cells showed half the index of oxidation compared to control (Figure 3B). Further evaluation revealed a profound defect in H_2_O_2_ release upon zymosan and *S. aureus* stimulation, consistent with the cellular phenotype of p40*^phox^* deficiency (Figure 3C). Moreover, protein electrophoresis of neutrophil lysate from the patient confirmed a reduction of approximately 50% in p40*^phox^* expression compared to controls, while the other NADPH subunits (gp91*^phox^*, p67*^phox^*, p47*^phox^*, and p22*^phox^*) exhibited expression more similar to healthy individuals (Figure 4). These data confirm that this patient with pSLE exhibited the cellular phenotype of p40*^phox^* deficient patients with affected p40*^phox^* protein expression, impaired ROS production after stimuli, and reduced activity detected by DHR.

## Conclusions and discussion

p40*^phox^* deficiency is a rare inborn error of immunity resulting from impaired but not abolished ROS production by the NADPH oxidase complex in phagocytic cells. The residual ROS production confers protection against invasive infections, but p40*^phox^* deficient patients can suffer from immune dysregulation. The most common inflammatory manifestations seen in p40*^phox^* patients are IBD and cutaneous inflammation consistent with lupus, including discoid lupus (1, 2). Defects in ROS production have been linked to autoimmune susceptibility, including lupus (3, 4).

pSLE is a severe early-onset autoimmune disease associated with positive autoantibodies and a broad clinical spectrum that can include fevers, rashes, arthritis, autoimmune cytopenias, and nephritis, among other features (6). Some patients with pSLE exhibit defects in genes associated with complement deficiencies, nucleic acid sensing, and B cell dysregulation, but previous studies have not confirmed defects in ROS production as a potential etiology of monogenic lupus (8).

To our knowledge this is the first case of pSLE associated with p40*^phox^* deficiency. While some p40*^phox^* deficient individuals have had lupus-like cutaneous lesions and ITP has been described (1, 2), our patient had an early-onset phenotype fulfilling classification criteria for SLE with multiple positive autoantibodies and no IBD manifestations to date.

Our patient's refractory ITP requires serial rituximab treatment for steroid-sparing purposes, which suggests that some of her autoimmunity is B-cell mediated. Epstein Barr virus transformed B cells from p40*^phox^* deficient patients exhibit severe impairments in ROS production (1). Little is known about the specific role of ROS in B cells and any contribution they may make to the development of autoimmunity. Further studies regarding defective ROS production in B cells are needed to decipher the pathophysiology of p40*^phox^* deficiency and the connection between ROS production and autoimmunity.

In summary, this case expands the clinical spectrum of p40*^phox^* deficiency and highlights the importance of considering this genetic disorder in the differential diagnosis of unresolved pSLE cases, particularly those of pre-pubertal onset and/or associated with recurrent infections. It also suggests DHR assays with index quantification may be relevant during initial evaluations of such patients. Finally, this case confirms the pivotal role of ROS production in immune regulation.

## Data Availability

The raw data supporting the conclusions of this article will be made available by the authors, without undue reservation.

## Supplementary material

Supplementary Table S1

Clinical course of p40*^phox^* deficient patient with pSLE.
