# Supplementary material for: Case Report: p40phox deficiency underlying pediatric-onset systemic lupus erythematosus
Source: Front Pediatr. 2024 Aug 20;12:1425874. doi: 10.3389/fped.2024.1425874 (PMC11368735; doi:10.3389/fped.2024.1425874)
Supplement: Supplementary file 1 [file Table1.docx]

# **Supplementary Material**

**Table**. Clinical course of p40*^phox^* deficient patient with pSLE.

| **Clinical presentation** | **Age of Onset** | **Treatment** |
| --- | --- | --- |
| Malar rash  Purpuric, vasculitic rash  Daily fevers for 2 weeks  Jaw pain  Autoimmune hemolytic anemia  Hypocomplementemia  Elevated ANA titer  Positive lupus anticoagulant | 7 years | Methylprednisolone  Hydroxychloroquine  Mycophenolate mofetil  Prednisone |
| Suspected herpes simplex virus blepharitis | 8 years | Antiviral |
| Granulomatous skin lesions  Urinary tract infections caused by *Klebsiella* *pneumoniae* and *E. coli*  Consolidative pneumonia with effusion (suspected *Streptococcus pneumoniae*)  Posterior reversible encephalopathy syndrome | 11 years | Rituximab  Antimicrobials |
| Hypergammaglobulinemia  Immune thrombocytopenia  +Smith antibodies/+ribonucleoprotein antibodies  *C. glabrata* urinary tract infection | 12 years | Rituximab  Mycophenolate mofetil  Azathioprine  Prednisone |
| Immune thrombocytopenia recurrence | 14 years | Rituximab |
| Immune thrombocytopenia recurrence | 16 years | Rituximab |
| Urinary tract infection caused by *E. faecalis* with presumed sepsis and shock  *C. albicans* urinary tract infection | 17 years | Antimicrobials  Prednisone |
